# Cardiovascular Risk Stratification and Statin Eligibility Based on the Brazilian vs. North American Guidelines on Blood Cholesterol Management

**DOI:** 10.5935/abc.20170088

**Published:** 2017-06

**Authors:** Fernando Henpin Yue Cesena, Antonio Gabriele Laurinavicius, Viviane A. Valente, Raquel D. Conceição, Raul D. Santos, Marcio S. Bittencourt

**Affiliations:** 1 Hospital Israelita Albert Einstein, São Paulo, SP - Brazil; 2 Instituto do Coração (InCor) - Faculdade de Medicina da Universidade de São Paulo, São Paulo, SP - Brazil; 3 Hospital Universitário da Universidade de São Paulo; São Paulo, SP - Brazil

**Keywords:** Cardiovascular Diseases, Cholesterol, Anticholesterelemic Agents, Risk Assessment, Hydroxymethylglutaryl-CoA Reductases, Practice Guidelines as Topic

## Abstract

**Background:**

The best way to select individuals for lipid-lowering treatment in the population is controversial.

**Objective:**

In healthy individuals in primary prevention:

1. to assess the relationship between cardiovascular risk categorized according to the V Brazilian Guideline on Dyslipidemia and the risk calculated by the pooled cohort equations (PCE);
2. to compare the proportion of individuals eligible for statins, according to different criteria.

**Methods:**

In individuals aged 40-75 years consecutively submitted to routine health assessment at one single center, four criteria of eligibility for statin were defined: BR-1, BR-2 (LDL-c above or at least 30 mg/dL above the goal recommended by the Brazilian Guideline, respectively), USA-1 and USA-2 (10-year risk estimated by the PCE ≥ 5.0% or ≥ 7.5%, respectively).

**Results:**

The final sample consisted of 13,947 individuals (48 ± 6 years, 71% men). Most individuals at intermediate or high risk based on the V Brazilian Guideline had a low risk calculated by the PCE, and more than 70% of those who were considered at high risk had this categorization because of the presence of aggravating factors. Among women, 24%, 17%, 4% and 2% were eligible for statin use according to the BR-1, BR-2, USA-1 and USA-2 criteria, respectively (p < 0.01). The respective figures for men were 75%, 58%, 31% and 17% (p < 0.01). Eighty-five percent of women and 60% of men who were eligible for statin based on the BR-1 criterion would not be candidates for statin based on the USA-1 criterion.

**Conclusions:**

As compared to the North American Guideline, the V Brazilian Guideline considers a substantially higher proportion of the population as eligible for statin use in primary prevention. This results from discrepancies between the risk stratified by the Brazilian Guideline and that calculated by the PCE, particularly because of the risk reclassification based on aggravating factors.

## Introduction

Although the relationship between the reduction in serum low-density lipoprotein cholesterol (LDL-c) levels and the reduction in cardiovascular events is indisputable,^1^ the best way to select individuals in the population for treatment with lipid-lowering drugs is controversial, and the recommendations vary in different guidelines.^2-7^

The V Brazilian Guideline on Dyslipidemia and Atherosclerosis Prevention (V Brazilian Guideline), published in 2013, is based on the classical precept, used for many years, of establishing more aggressive LDL-c goals for individuals at higher cardiovascular risk.^2^

The American College of Cardiology (ACC)/American Heart Association (AHA) guideline, from now on referred to as North American Guideline, also published in 2013, does not advocate meeting LDL-c goals, but elects groups of individuals who benefit from statin use, based on their clinical antecedents or absolute risk for major cardiovascular events.^3^ In addition, the North American Guideline proposes new equations to calculate the cardiovascular risk, the pooled cohort equations (PCE), derived from cohorts representative of the North American population.^8^

Both the way of stratifying the cardiovascular risk and the criteria for statin eligibility can vary substantially, depending on the guideline used, which impacts the individual therapeutic decision-making and has an expressive repercussion to the health system.

The objectives of this study, carried out with mainly healthy individuals in primary prevention and with no clinical manifestation indicative of high cardiovascular risk, were:

1. to assess the relationship between cardiovascular risk categorized according to the V Brazilian Guideline recommendations and the risk calculated by use of the PCE;
2. to compare the proportion of individuals eligible for statins, according to either the V Brazilian Guideline or the North American Guideline criteria.

## Methods

### Population studied

The present study included individuals consecutively evaluated at the Preventive Medicine Center of the Albert Einstein Israeli Hospital (São Paulo-SP) from 01/2009 to 12/2015. Data were prospectively collected. The study protocol comprises complete clinical history and physical examination performed by a clinician, treadmill exercise test and blood tests (lipid profile, fasting glycemia, high-sensitivity C-reactive protein [hs-CRP]), as previously detailed.^9^

Individuals with the following characteristics were excluded: age < 40 years or > 75 years; self-reported antecedents or detection of significant clinical or subclinical cardiovascular atherosclerotic disease, abdominal aortic aneurysm or diabetes mellitus; LDL-c ≥ 190 mg/dL; and current use of lipid-lowering drugs. In addition, individuals with parameters outside the recommended range for using the cardiovascular risk equations (total cholesterol < 130 mg/dL or > 320 mg/dL, high-density lipoprotein cholesterol [HDL-c] < 20 mg/dL or > 100 mg/dL, systolic blood pressure < 90 mm Hg or > 200 mm Hg) were excluded, as were those whose missing data prevented risk calculation.

### Cardiovascular risk according to the V Brazilian Guideline

As recommended by the V Brazilian Guideline, the Framingham general cardiovascular risk score was calculated by using the proper equation with continuous variables (age, systolic blood pressure, total cholesterol, HDL-c) and categorical variables (sex, arterial hypertension treatment or non-treatment, presence or absence of diabetes mellitus and smoking).^10^ That score calculates the risk of death from coronary artery disease, myocardial infarction, angina, ischemic or hemorrhagic stroke, transient ischemic attack, peripheral vascular disease or heart failure in 10 years.^10^

In addition, the presence or absence of aggravating risk factors, capable of re-stratifying cardiovascular risk, based on the V Brazilian Guideline recommendations, was assessed.^2^ The following aggravating risk factors were considered: hs-CRP > 2 mg/L and < 10 mg/L in the absence of inflammatory conditions (not related to atherosclerosis); family history of premature coronary artery disease (male first-degree relative < 55 years or female first-degree relative < 65 years); metabolic syndrome (according to the *International Diabetes Federation* criteria^11^); and subclinical atherosclerosis (detected on ultrasound of the carotid arteries or computed tomography of the coronary arteries).^2^ The assessment of subclinical atherosclerosis is not part of the routine protocol at our service, so its request was up to the clinician in charge or to the patient’s attending physician.

Individuals with a Framingham general cardiovascular risk score < 5% were considered at low or intermediate risk, depending on the absence or presence of a family history of premature coronary artery disease, respectively. Women with a general risk score between 5% and 10%, as well as men with a general risk score between 5% and 20%, were classified as at an intermediate or high risk, depending on the absence or presence of aggravating factors, respectively. Women and men with global risk scores > 10% and > 20%, respectively, were stratified as at high risk.^2^

### Cardiovascular risk according to the PCE

The cardiovascular risk was also calculated by use of the PCE, as recommended by the North American Guideline.^3,8^ The PCE used a more modern statistical modeling that allows greater flexibility in accommodating the clinical variables used for risk prediction, which are the same described above for the Framingham general risk score, in addition to ethnicity.^8^ Differently from the general risk score, the PCE calculate the risk of major cardiovascular events, such as death from coronary artery disease, non-fatal myocardial infarction and fatal or non-fatal stroke, in 10 years.^8^

### Statin eligibility criteria

Based on the V Brazilian Guideline, two criteria of eligibility for statin use were arbitrarily considered: LDL-c above the goal advocated by the V Brazilian Guideline (BR-1 criterion) or LDL-c at least 30 mg/dL above that goal (BR-2 criterion).

The following LDL-c goals are recommended by the V Brazilian Guideline: < 100 mg/dL for individuals at intermediate risk and < 70 mg/dL for those at high risk.^2^ Individuals at low cardiovascular risk, according to the V Brazilian Guideline, to whom the guideline recommends an individualized LDL-c goal, were not considered eligible for statin use according to the BR-1 and BR-2 criteria.

According to the North American Guideline, statin should be considered for individuals aged between 40 and 75 years, not diagnosed with clinical atherosclerotic cardiovascular disease or diabetes mellitus, with LDL-c between 70 mg/dL and 189 mg/dL, and cardiovascular risk by using PCE ≥ 7.5% in 10 years. Those with risk between 5.0% and < 7.5% can also be considered for statin use.^3^

Thus, this study considered two criteria of eligibility for statin use based on the North American Guideline: cardiovascular risk by using the PCE ≥ 5.0% (USA-1 criterion) or ≥ 7.5% (USA-2 criterion).

### Statistical analysis

Knowing in advance that the data bank used in this study is mainly composed of male individuals and does not represent the general Brazilian population, the cardiovascular risk stratification was planned to be evaluated separately for women and men. Likewise, statin eligibility was analyzed in subgroups defined by sex, age group and cardiovascular risk categories.

Categorical variables were expressed as percentages, and the chi-square test was used for comparisons. Continuous variables were expressed as means and standard deviations; non-paired Student *t* test was used to compare baseline characteristics between men and women, while analysis of variance (ANOVA) was used to compare the cardiovascular risk obtained from the PCE among the low, intermediate and high risk categories. Considering the large sample size and the central limit theorem, according to which the distribution of the sample means always tends to normality, we assumed that all variables had a normal distribution and could be analyzed by use of parametric tests.

The analyses were performed with Microsoft Office Excel tools and Stata statistical program, 13.0 version. A p value < 0.05 was considered statistically significant.

### Ethical aspects

The study was approved by the Ethics Committee in Research of the Albert Einstein Israeli Hospital (CAAE 54537916.2.0000.0071). Considering that this is a retrospective study using a data bank and involving a large number of individuals, many of whom seen several years before this study began, the written informed consent could not be used and the Ethics Committee approved its waiver.

## Results

### Population studied and its characteristics

**Figure 1:**
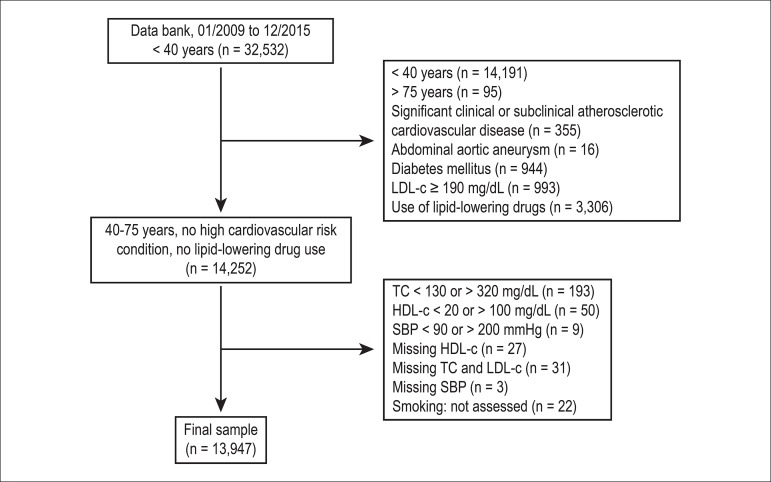
Flowchart detailing individuals included in and excluded from the study. SBP: systolic blood pressure; TC: total cholesterol.

Figure 1 details the individuals included in and excluded from the study. From the 32,532 individuals initially identified in the data bank, 18,585 (57%) were excluded, most of whom (76%) because of age < 40 years.

Of the final sample of 13,947 individuals, 9,901 (71%) were male. Table 1 shows the main characteristics of the population studied. Most women were at low cardiovascular risk. Despite the comparable mean age, the male population was characterized by a less favorable lipid profile, higher frequency of metabolic syndrome-related changes and higher cardiovascular risk as compared to women.

**Table 1:** Characteristics of the study population

		Total (n = 13,947)	Women (n = 4,046)	Men (n = 9,901)	p (women vs men)
Age (years)		48 ± 6	48 ± 6	48 ± 7	< 0.01
BMI (kg/m^2^)		26.8 ± 4.2	25.3 ± 4.5	27.5 ± 3.9	< 0.01
Total cholesterol (mg/dL)		203 ± 31	198 ± 31	205 ± 31	< 0.01
LDL-c (mg/dL)		127 ± 28	119 ± 28	130 ± 28	< 0.01
HDL-c (mg/dL)		49 ± 13	58 ± 14	45 ± 11	< 0.01
Triglycerides (mg/dL)		137 ± 85	106 ± 57	150 ± 91	< 0.01
Fasting glycemia (mg/dL)		89 ± 11	85 ± 9	90 ± 11	< 0.01
hs-CRP (mg/L)*		2.7 ± 5.5	3.1 ± 5.9	2.5 ± 5.3	< 0.01
Arterial hypertension		2,117 (15)	419 (10)	1,698 (17)	< 0.01
Metabolic syndrome		3,557 (26)	613 (15)	2,944 (30)	< 0.01
Smoking		1,268 (9)	335 (8)	933 (9)	0.04
Family history of premature coronary disease		1,399 (10)	432 (11)	967 (10)	< 0.11
Cardiovascular risk (V Brazilian Guideline)	Low	5,049 (36)	2,969 (73)	2,080 (21)	< 0.01
	Intermediate	4,352 (31)	577 (14)	3,775 (38)	
	High	4,546 (33)	500 (12)	4,046 (41)	
Framingham general cardiovascular risk (% in 10 years)		8.0 ± 6.7	3.5 ± 2.8	9.8 ± 7.0	< 0.01
Cardiovascular risk (PCE, ACC/AHA 2013, % in 10 years)		3.7 ± 4.1	1.4 ± 1.8	4.6 ± 4.3	< 0.01

Data expressed as mean ± standard deviation or n (%). ACC/AHA: American College of Cardiology/American Heart Association; BMI: body mass index; hs‑CRP: hogh‑sensitivity C reactive protein; PCE: pooled cohort equations.

* Data on hs-CRP were available in 96% of the study participants.

A significant percentage of individuals was re-stratified into a higher-risk category because of the presence of an aggravating factor. Of the 577 women at intermediate risk based on the V Brazilian Guideline, 332 (58%) had a Framingham general risk score < 5% and family history of premature coronary artery disease. However, that situation occurred in only 187 (5%) of the 3,775 men stratified as at intermediate risk.

**Figure 2:**
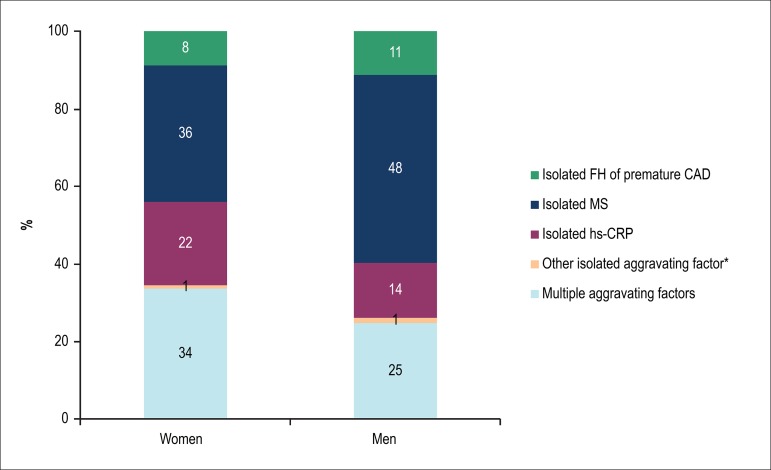
Aggravating cardiovascular risk factors responsible for risk re-stratifcation from intermediate to high risk. CAD: coronary artery disease; FH: family history; hs-CRP: high-sensitivity C-reactive protein; MS: metabolic syndrome. * Albuminuria, left ventricular hypertrophy, carotid intima-media thickness or coronary calcifcation.

In addition, of the 500 women at high risk according to the V Brazilian Guideline, 366 (73%) had a Framingham general risk score between 5% and 10%, and were re-stratified due to the presence of an aggravating factor. Of the 4,046 men at high risk, 3,221 (80%) had a Framingham general risk score between 5% and 20% and an aggravating factor. Metabolic syndrome was the major single aggravating factor responsible for re-stratification into high risk, for both sexes (Figure 2).

### Cardiovascular risk by the V Brazilian Guideline versus risk calculated by the PCE

**Figure 3:**
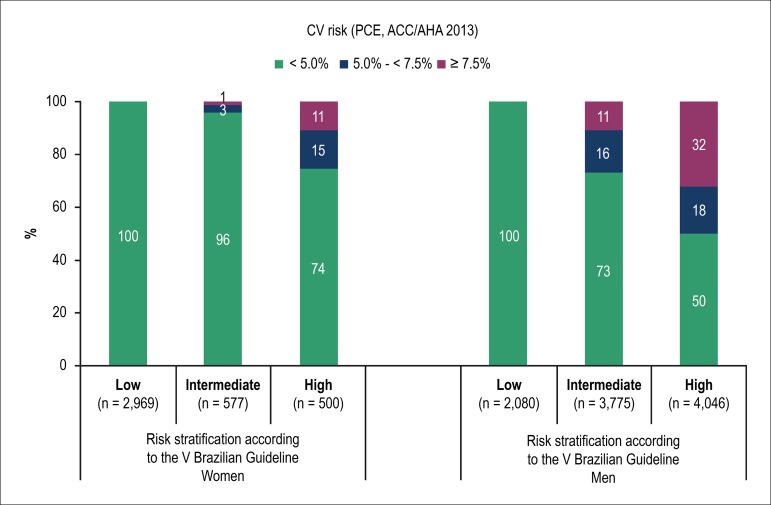
Categories of cardiovascular (CV) risk based on the pooled cohort equations (PCE) (ACC/AHA 2013), by sex and CV risk category according to the V Brazilian Guideline.

Figure 3 shows the distribution of the cardiovascular risk categories calculated by using the PCE, according to the stratum of cardiovascular risk determined by the V Brazilian Guideline. For both sexes, a high proportion of individuals with PCE risk < 5% in 10 years was observed, even in the categories of intermediate and high risk, according to the V Brazilian Guideline. However, only a minority of individuals stratified as at high risk, according to the V Brazilian Guideline, had a PCE risk ≥ 7.5% in 10 years.

Among women, the means ± standard deviations of cardiovascular risks according to the PCE were as follows: 0.8 ± 0.6% in the low-risk category; 1.8 ± 1.6% in the intermediate-risk category; and 4.3± 3.4% in the high-risk category (p < 0.01). Among men, the respective values were 1.2 ± 0.4%, 4.1 ± 2.4% and 6.9 ± 5.4% (p < 0.01).

### Statin eligibility

Statin eligibility was significantly higher according to the BR-1 and BR-2 criteria, as compared to the USA-1 and USA-2 criteria, for both women and men. According to the BR-1, BR-2, USA-1 and USA-2 criteria, 975 (24%), 705 (17%), 156 (4%) and 63 (2%) women, respectively, would be eligible for statin use (p < 0.01). The respective numbers for men were 7,381 (75%), 5,704 (58%), 3,050 (31%) and 1,696 (17%, p < 0.01).

**Figure 4:**
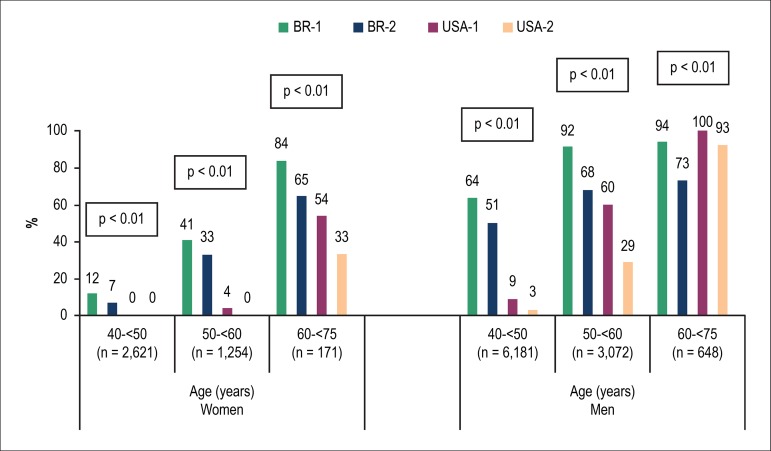
Proportion of individuals eligible for statin based on different criteria, by sex and age group.

**Figure 5:**
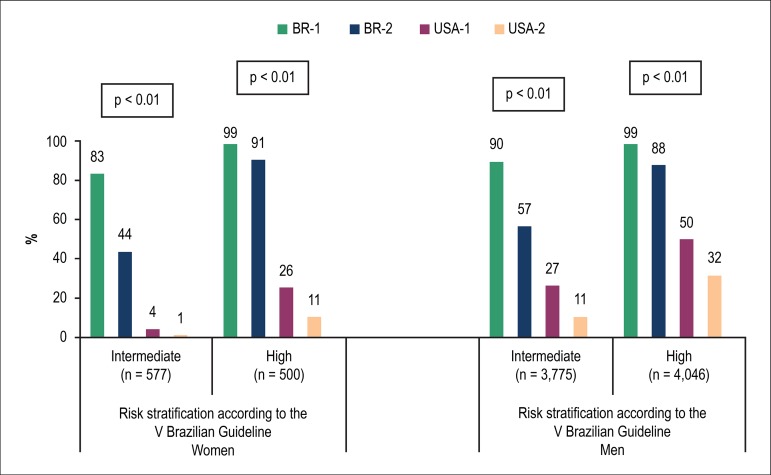
Proportion of individuals eligible for statin based on different criteria, by sex and cardiovascular risk according to the V Brazilian Guideline.

A higher proportion of women eligible for statins according to the V Brazilian Guideline criteria as compared to those of the North American Guideline was observed in all age groups analyzed, and in those both at intermediate and high risks, according to the V Brazilian Guideline (Figures 4 and 5). The proportion of candidates for statin was 10 times greater according to the BR-1 criterion, as compared to the USA-1 criterion, for women aged between 50 and < 60 years (Figure 4), 19 times greater in those classified as at intermediate risk according to the V Brazilian Guideline, and 4 times greater in those at high risk (Figure 5).

In men, the higher rate of statin eligibility according to the Brazilian criteria was also observed in those at intermediate risk and at high risk (Figure 5) and aged < 60 years, but this was not detected in the subgroup aged 60-75 years (Figure 4). As compared to the USA-1 criterion, statin eligibility according to the BR-1 criterion increases by 7 times in men aged between 40 and < 50 years (Figure 4), triples in those at intermediate risk, and doubles in those at high risk (Figure 5).

### Agreement and disagreement between the statin eligibility criteria

The BR-1 and USA-1 criteria were used to assess agreement and disagreement regarding statin eligibility based on the Brazilian and North American guidelines.

Among women, there was agreement between the criteria to not indicate statin in 76% of the population, while both criteria considered statin in only 4% of the cases.

Among men, there was agreement between the criteria in 54% of the cases: in 24% statin would not be considered by any criterion, while 30% of the individuals would be candidates for statin according to both criteria.

**Figure 6:**
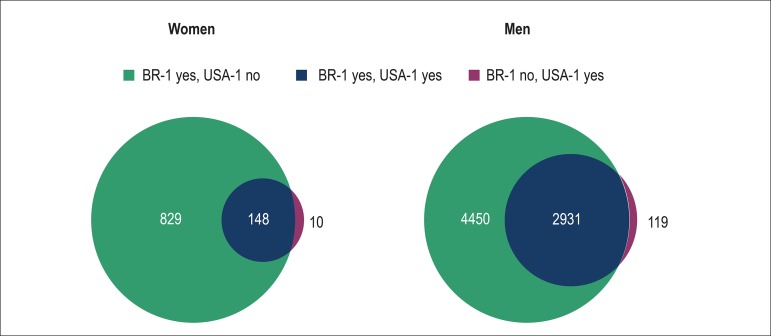
Venn diagram showing the number of eligible (“yes”) or non-eligible (“no”) individuals for statin use based on the BR-1 and USA-1 criteria, by sex.

**Figure 7:**
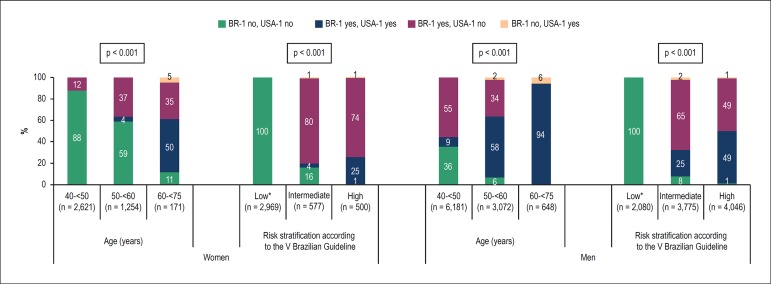
Proportion of eligible (“yes”) or non-eligible (“no”) individuals for statin use based on the BR-1 and USA-1 criteria, by sex, age group and cardiovascular risk according to the V Brazilian Guideline. *Individuals classifed as at low risk based on the V Brazilian Guideline were considered non-eligible for statin use according to the BR-1 criterion (see Methods).

Eighty-five percent of women and 60% of men who were eligible for statin based on the BR-1 criterion would not be candidates for statin based on the USA-1 criterion (Figure 6). However, almost all individuals eligible for statin use based on that North American criterion would also be eligible based on that Brazilian criterion (Figure 6). The rare cases eligible for statin based on the USA-1 criterion, but not on the BR-1 criterion, were mainly observed among the elderly (Figure 7).

Analyzing the subgroups defined by age group, the disagreement rate between the BR-1 and USA-1 criteria increases with age in women, but decreases in men (Figure 7). While for most (88%) women between 40 and < 50 years there was agreement regarding the non-indication for statin, for men of the same age group there was 50% disagreement between the criteria (Figure 7). However, while the criteria agreed in considering statin for 94% of the men aged 60-75 years, for women of the same age group disagreement between the criteria reached 40% (Figure 7).

Among individuals classified as at intermediate risk and, to a lower extent, at high risk according to the V Brazilian Guideline, the disagreement rate between the BR-1 and USA-1 criteria was high, with an expressive number of cases of statin eligibility by the BR-1 criterion, but not by the USA-1 criterion, mainly among women (Figure 7).

## Discussion

In the present study, we observed a large discrepancy in statin eligibility between the V Brazilian Guideline and the 2013 ACC/AHA Cholesterol Guideline, the number of candidates for statin being significantly higher following the recommendations of the Brazilian Guideline.

Among individuals stratified as at intermediate or high risk, according to the V Brazilian Guideline, the number of those eligible for statin based on the Brazilian Guideline, but not on the North American Guideline, is high mainly among women. This is directly related to the fact that most individuals considered at intermediate or high risk by the V Brazilian Guideline has a low risk calculated with the PCE. For those classified as at high risk according to the Brazilian Guideline, for example, the mean risk in 10 years calculated with the PCE was < 5% for women and < 7% for men, while North American guidelines consider individuals at high risk those with risk ≥ 15% or ≥ 20% in 10 years.^4,12^

That discrepancy between the risk stratifications recommended by the V Brazilian Guideline and the North American Guideline is associated with the finding that most individuals classified as at high risk by the V Brazilian Guideline has a Framingham general risk score at intermediate levels, being re-stratified due to the presence of an aggravating factor, mainly metabolic syndrome and hs-CRP elevated levels.

The magnitude of risk reclassification observed in this study might be overestimated as compared to that of clinical practice. The hs-CRP measurement was performed as part of this study protocol and was available in 96% of the participants, a proportion certainly higher than that in the real world. In addition, hs-CRP was measured only once. Among individuals reclassified due to hs-CRP elevation, there might be cases in which that elevation would not repeat, if a second measurement was performed, and cases in which the hs-CRP increase occurred due to incipient or subclinical inflammatory conditions, not diagnosed or not reported by the attending physician.

The highest rate of statin eligibility according to the Brazilian Guideline as compared to the North American Guideline can also be related to changes in the V Brazilian Guideline^2^ as compared to the previous one,^13^ which made it more “aggressive”: a reduction in the LDL-c goals, a reduction in the thresholds to categorize intermediate and high risks (mainly in women), and the adoption of the Framingham general risk score in the place of the risk score for “hard” coronary outcomes. The Canadian guideline, for example, which also recommends risk stratification based on the same general cardiovascular risk score, although modified (the risk is doubled in the presence of family history of premature cardiovascular disease), uses higher cutoff points than those of the V Brazilian Guideline to separate the risk categories: low-risk individuals are those with score < 10%, intermediate-risk individuals are those with score ≥ 10% and < 20%, and high-risk individuals are those with score ≥ 20% in 10 years, with no distinction between men and women.^6^

Our results differ from those of a recent publication that reports a higher number of candidates for statin according to the North American Guideline, as compared to the IV Brazilian Guideline on Dyslipidemia,^13^ in participants of the ELSA-Brasil Study.^14^ The North American recommendations have also shown higher statin eligibility as compared to the European guidelines,^15,16^ but not to the Canadian guideline.^17^

The only subgroup analyzed in this study that showed a high agreement between the Brazilian and North American criteria was that of men aged 60-75 years, whose proportion of statin eligibility was very elevated, regardless of the criterion used. Other analyses have also detected a high rate of statin eligibility for the elderly, when applying the North American Guideline.^16^ In addition, that finding might be related to the possibility that PCE overestimate the cardiovascular risk in the subgroups of higher risk, such as the elderly, which has been reported in some cohorts.^18,19^

More individuals on statins would mean a lower mean LDL-c level and greater cardiovascular benefit for the population, because of the unquestionable relationship between those two factors, even in populations at lower cardiovascular risk.^20^ That benefit, however, would be provided at the expense of higher costs, higher incidence of statin-related side effects, and especially a greater number needed to treat (NNT) to prevent one cardiovascular event, which foster discussions on medical overtreatment.^21^ Cost-effectiveness analyses might help to better define the advantages of following one or the other guideline.

### Limitations

This study was based on theoretical considerations that might not reflect precisely the real world. For example, this study considered non-eligible for statin those stratified as at low cardiovascular risk, according to the V Brazilian Guideline, but part of those individuals could receive a drug prescription in clinical practice. Conversely the present study did not include the North American Guideline recommendation to consider statin use for individuals with a low calculated cardiovascular risk, but with some conditions known to increase the risk, such as LDL-c ≥ 160 mg/dL, family history of premature atherosclerotic cardiovascular disease, hs-CRP elevation, and significant coronary calcification on computed tomography.^3^

## Conclusions

For healthy individuals in primary prevention, management of blood cholesterol based on the V Brazilian Guideline on Dyslipidemias or the 2013 ACC/AHA Cholesterol Guideline can vary substantially. Among those classified as at intermediate or high risk according to the V Brazilian Guideline, there is a high proportion of individuals eligible for statin according to the Brazilian Guideline criteria, but not according to the North American Guideline criteria. This finding is associated with the fact that most individuals at intermediate or high risk according to the Brazilian Guideline have a low risk calculated by the PCE, in addition to the fact that most individuals classified as at high risk owe that to the presence of an aggravating factor.

Our results can allow a critical reflection on the current guidelines and continuous improvement of the recommendations. In addition, they can help attending physicians with clinical judgement and therapeutic decision making.
